# CTG18.1 expansion in transcription factor 4 (*TCF4*) in corneal graft failure: preliminary study

**DOI:** 10.1007/s10561-023-10123-y

**Published:** 2024-01-11

**Authors:** Ida Maria Westin, Andreas Viberg, Irina Golovleva, Berit Byström

**Affiliations:** 1 Department of Medical Biosciences, Medical and Clinical Genetics, Umeå University, Umeå, Sweden; 2 Department of Clinical Sciences, Ophthalmology, Umeå University, Umeå, Sweden

**Keywords:** Fuchs endothelial corneal dystrophy, Transcription factor 4, *TCF4*, CTG18.1, Descemet stripping automated keratoplasty, DSAEK

## Abstract

Fuchs endothelial corneal dystrophy (FECD) is caused by a corneal endothelial cell loss, leading to corneal edema and visual impairment. The most significant genetic risk factor for FECD is an expansion of the CTG18.1 locus in transcription factor 4 (*TCF4*). The current treatment for severe FECD is corneal transplantation, with Descemet stripping automated keratoplasty (DSAEK) as a common surgical method. Although successful in most cases, the risk for transplant failure due to diverse causes must be considered. In this study, we investigated if presence of *TCF4* CTG18.1 expansion with more than 31 (n ≥ 31) repeats in donated corneal grafts could be a reason for corneal transplant failure after DSAEK. For this, nine consecutively failed DSAEK corneal grafts were genotyped for CTG18.1 repeat length. One-sided Mann–Whitney U test was performed to evaluate if failed DSAEK corneal grafts had longer CTG18.1 repeats than healthy controls from the same population. All failed corneal grafts had CTG18.1 n ≤ 27 with a median of 18 (IQR 8.0) repeats for the longest allele. There was no statistical difference in CTG18.1 repeat lengths between failed corneal grafts and the geographically matched healthy control group. In conclusion, none of the nine failed corneal grafts in our material had CTG18.1 repeat lengths ≥ 31, a cut-off known to have a biological relevance in FECD. Thus, our results suggest that the assessment of donors and inspection of the corneal tissue before the decision for procurement is sufficient, in terms of recognizing FECD in the donor.

## Introduction

Fuchs endothelial corneal dystrophy (FECD) is a disease of the innermost layer of the cornea, the endothelium, affecting both eyes in ~ 3–11% of the elderly population (Lorenzetti et al. 1967; Zoega et al. 2006; Higa et al. 2011; Eghrari et al. 2012, 2015). In FECD, there is a gradual loss of corneal endothelial cells (CECs) and the underlying Descemet’s membrane, which is composed of extracellular matrix, starts to bulge out between the remaining CECs, that can be visualized with specular microscopy as corneal guttata (Eghrari et al. 2015). In some individuals, the disease progresses from corneal guttata to corneal edema due to impaired corneal endothelial pump function, that results in visual disability, and in severe cases painful corneal epithelial blisters (Eghrari et al. 2015; Viberg et al. 2022).

FECD has been linked to a triplet repeat expansion in Transcription Factor 4 (*TCF4*) denoted as CTG18.1 (Wieben et al. 2012), where an expansion over 40 (Mootha et al. 2014; Xing et al. 2014; Kuot et al. 2017; Skorodumova et al. 2018) or 50 repeats (Nanda et al. 2014; Nakano et al. 2015; Foja et al. 2017; Rao et al. 2017; Zarouchlioti et al. 2018; Viberg et al. 2022) is regarded as a major risk factor for developing FECD. Presence of toxic RNA foci in corneal endothelial cells formed of transcribed *TCF4* RNA from an expanded CTG18.1 allele (n ≥ 31) is dependent on the length of both CTG18.1 alleles and considered as one of the many pathological mechanisms in FECD (Du et al. 2015; Mootha et al. 2015; Hu et al. 2018; Zarouchlioti et al. 2018; Rong et al. 2019).

The only curative treatment for severe FECD is a corneal transplantation, and FECD is currently one of the most common indications for corneal transplantation in North America and Europe (Matthaei et al. 2017). In Sweden, endothelial keratoplasty, i.e. Descemet membrane endothelial keratoplasty (DMEK) or Descemet stripping automated endothelial keratoplasty (DSAEK), are the general methods used for corneal transplantation in diseases of the corneal endothelial layer. The risk of graft failure in in FECD cases after DSAEK is ~ 5% within 2 years after surgery (Hjortdal et al. 2013). Graft failure has been associated with high donor age, unexperienced surgeons, different preparation techniques for donor tissue, donors with conjunctival hyperemia (Kanavi et al. 2020) and immunological rejection (Gómez-Benlloch et al. 2021). However, the reason for corneal endothelial decompensation after DSAEK is not always known, especially not when there is a primary graft failure or problems for the graft to attach after DSAEK. During the donation process, each corneal tissue is evaluated to reveal possible endothelial disease, which is a contraindication for corneal graft donation, but no evaluation of the triplet repeat expansion in *TCF4*, known to be associated with FECD is routinely carried out. We have recently shown a strong association between FECD and CTG18.1 expansion in *TCF4* in a Swedish cohort of FECD patients, and also demonstrated that ~ 4% of individuals from the general population are carriers of expanded CTG18.1 alleles (Viberg et al. 2022). We hypothesized whether an expansion of *TCF4* CTG18.1 in the donor tissue could be the cause for graft failure. To investigate this, we collected decompensated donor tissues at re-DSAEK surgery, and analyzed them for the presence of *TCF4* CTG18.1 expansion.

## Materials and methods

### Study design

In this study we investigated patients with severe FECD treated with DSAEK, for whom the transplant failed and led to re-DSAEK. During re-DSAEK, the failed grafts (originating from a donor cornea at first surgery) were collected and genotyped for *TCF4* CTG18.1. In cases with a result of mixed genotype from both the FECD patient (i.e., the recipient) and the donor (i.e., the failed graft), we distinguished between the donor’s and the patient’s *TCF4* CTG18.1 genotype by using DNA extracted from the failed corneal graft and DNA from peripheral blood of the corresponding recipient. Furthermore, results from this present study were compared to our previous *TCF4* CTG18.1 association study (Viberg et al. 2022). The study results on the *TCF4* CTG18.1 genotype in FECD patients and healthy controls from geographically matched Swedish population were used for comparison in this current study.

### Biological samples

Nine failed corneal grafts were included in this study, taken consecutively over three years during clinical re-DSAEK of FECD patients going through surgery. The median age of donors was 69 (IQR 14) years and the median time in days between the first DSAEK and re-DSAEK was 531 (IQR 1085) days (Table 1). Three out of the nine donors (33%) were females (Table 1). Blood samples were also attained by collecting peripheral blood in EDTA vacutainers from six of the FECD patients.

**Table 1:** Donor tissues used in the study from failed corneal grafts after Descemet stripping automated keratoplasty

	Donors (*n* = 9)
*Age in years*	
Median age (IQR)	69 (14)
Range age	25–89
*Sex*	
Female	3 (33%)
Male	6 (66%)
*Days until reDSAEK*	
Median days (IQR)	531 (1085)
Range days	77–2181
* TCF4 CTG18.1*	
> 40 Repeats	0 (0%)
Median repeats (IQR)	18 (8)
Range repeats	12–27

*IQR* Interquartile range

A control group encompassing 102 young healthy individuals from the geographically matched Swedish population and 85 FECD patients was also included for comparison (Viberg et al. 2022).

All tissues were handled anonymously, and the study was approved by the Swedish Ethical Review Authority (2019-01744) and performed according to the Tenets of the World Medical Association Declaration of Helsinki.

### DNA extraction

Failed corneal grafts were removed and immediately put in RNAlater (Invitrogen, Waltham, USA) and thereafter washed twice with phosphate-buffered-saline (PBS) and frozen in − 80 °C until the day of DNA extraction. The genomic DNA was extracted with NucleoSpin Tissue XS (Macherey–Nagel, Düren, Germany) and eluted in 20 µl buffer included in the kit. DNA concentration was measured with spectrophotometry on DeNovix DS-11 FX (DeNovix, Wilmington, USA).

Genomic DNA from blood samples was extracted with Puregene Blood Core Kit C (Qiagen, Hilden, Germany) and the DNA was eluted in 1X Tris–EDTA buffer.

### TCF4 CTG18.1 genotyping

The *TCF4* CTG18.1 repeat length was determined with short tandem repeat PCR (STR-PCR). 1–40 ng of genomic DNA was added in 25 µl of STR-PCR master mix containing 0.2 mM dNTP (Roche Diagnostics, Basel, Switzerland), 1X KAPA2G Buffer A (Roche, Basel, Switzerland), 1X KAPA Enhancer (Roche, Basel, Switzerland), 0.48 µM forward primer (5′- 6FAM-AAATCCAAACCGCCTTCCAA -3′), 0.48 µM reverse primer (5′- AATGCACACCTTCCCTGAGT -3′) and 0.5 U KAPA2G Robust HotStart DNA polymerase (Roche, Basel, Switzerland). PCR products were separated on the ABI3500 Dx genetic analyzer instrument (Applied Biosystems, Waltham, Massachusetts, USA), and the raw data was analyzed using GeneMapper Software 5 (Applied Biosystems). If electropherogram peaks were too high, the PCR products were diluted 20–40X in pH_2_O prior to separation on the instrument. Samples having only one peak on the electropherogram were analyzed with triplet repeat primed PCR (TP-PCR) as described elsewhere (Viberg et al. 2022). In the current study a cut-off of n ≥ 31 for CTG18.1 was considered as an expanded allele. Individuals from the control group (n = 102) and FECD patients (n = 85) were genotyped previously and the results were described by Viberg et al. (2022).

### Statistical analysis

Interquartile range (IQR) was calculated to determine the distribution of data. One-sided Mann–Whitney U test was used to compare the longest CTG18.1 alleles in the group of failed corneal grafts after DSAEK (n = 9) against the longest alleles in the control group of young individuals from the Swedish population (n = 102) (Viberg et al. 2022).

## Results

Nine failed DSAEK corneal grafts were genotyped for presence of *TCF4* CTG18.1 expansion. Three of the grafts had four peaks on the electropherogram, indicating presence of both patient’s and donor’s alleles. For these specimens, blood samples were available from the FECD patients, and therefore we could determine donor’s alleles by exclusion of patient’s alleles.

All failed corneal grafts had *TCF4* CTG18.1 alleles less than ≤ 27 repeats, with a median of 18 (IQR 8.0) repeats for the longest alleles (Table 2).

**Table 2:** Repeat length of *TCF4* CTG18.1 in failed corneal grafts after DSAEK surgery

Donors	Allele 1	Allele 2
I	18	25
II	15	26
III	12	18
IV	15	18
V	12	27
VI	16	18
VII	18	26
VIII	12	15
IX	12	12

**Fig. 1:**
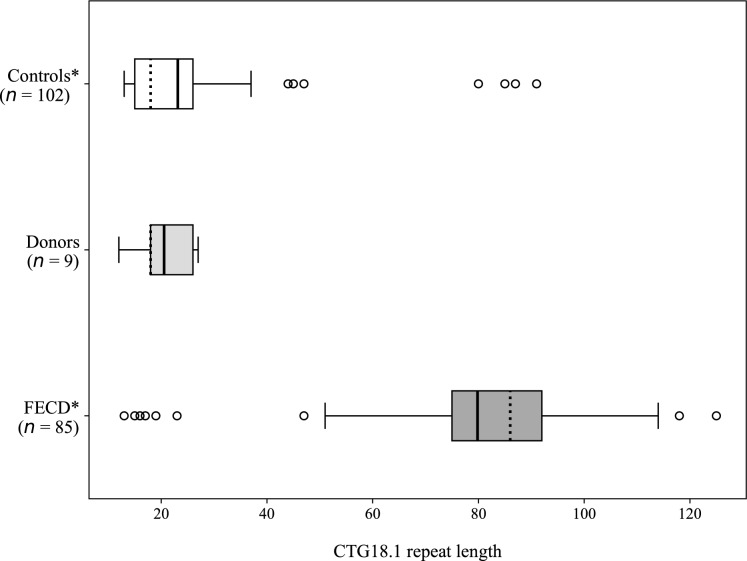
Distribution of the longest *TCF4* CTG18.1 alleles in controls including young individuals from the Swedish population (white) (Viberg et al. 2022), failed corneal grafts denoted as donors (light grey) and FECD patients from northern Sweden (dark grey) (Viberg et al. 2022). Filled vertical lines within boxes display means and dotted vertical lines within boxes display medians. Empty circles display outliers. *These individuals were genotyped in a previously published study (Viberg et al. 2022)

The longest alleles in the failed corneal grafts (n = 9) were statistically compared to the longest alleles in the control group of 102 individuals from the geographically matched population (Viberg et al. 2022). This control group had a median of 18 repeats (IQR 11.0) for the longest alleles (Fig. 1). The test came out as non-significant (p = 0.43, Mann–Whitney U). The longest alleles in the failed corneal grafts (n = 9) were also visually plotted with the longest alleles of 85 FECD patients (Fig. 1), who were genotyped in the previous study and had a median of 86 repeats (IQR 17.5) (Viberg et al. 2022).

## Discussion

Before a donor is approved for corneal graft donation a thorough checkup of both the donor and the corneal tissue is made in each tissue establishment and eye bank. The corneal tissue is visually screened for any abnormalities, and the cell density of the corneal endothelium is evaluated before the donated cornea can be procured. As of today, corneal donors are not genotyped for *TCF4* CTG18.1 in clinical routine prior to transplantation. Thus, it is not known if the donors carry an expanded *TCF4* CTG18.1 allele, i.e. a risk for development of FECD in the future, which might compromise the results of corneal transplantation especially in the context of corneal graft failure. This could above all be a risk when the donor is young and the features of FECD has not yet evolved.

In this study, we investigated if *TCF4* CTG18.1 expansion could be the cause of corneal graft failure after DSAEK surgery. A CTG18.1 allele with at least 31 repeats has previously been shown to produce toxic RNA foci in corneal endothelial cells, if the second allele is close to 30 repeats (Zarouchlioti et al. 2018). We found that none of the failed corneal grafts had CTG18.1 alleles with more than 27 repeats, with the second allele being 18 repeats at the longest, indicating that *TCF4* CTG18.1 is likely not the cause for corneal graft failure after DSAEK surgery in these cases.

The median repeat length of the longest alleles for the failed corneal grafts was compared to a control group of 102 individuals from the same ethnic population (Viberg et al. 2022). The test was conducted to rule if the donors had longer CTG18.1 alleles than the population control group, but this hypothesis was rejected.

In conclusion, our study suggests that the evaluation of donors and the checkup of the corneal tissue before the decision for procurement in the present eye bank are sufficient in terms of recognizing FECD in the donor. A limitation of our study is the few cases of failed corneal grafts, collected consecutive from re-DSAEK over three years, resulting in a scarce, though precious collection. Nevertheless, the data is of particular interest since the results after a re-transplantation are inferior and additional evaluation tools of the donated tissues are of great value to limit adverse outcome. Our results suggest that CTG18.1 expansion (n ≥ 31 repeats) in *TCF4* is not a common cause for graft failure, when examining grafts from our tissue bank. Still, *TCF4* CTG18.1 genotyping could be a tool for evaluating the suitability of corneal graft donors, in particular if the donor is young due to the late onset nature of FECD.

## Data Availability

All data generated or analysed during this study are included in this published article.
